# The ECOUTER methodology for stakeholder engagement in translational research

**DOI:** 10.1186/s12910-017-0167-z

**Published:** 2017-04-04

**Authors:** Madeleine J. Murtagh, Joel T. Minion, Andrew Turner, Rebecca C. Wilson, Mwenza Blell, Cynthia Ochieng, Barnaby Murtagh, Stephanie Roberts, Oliver W. Butters, Paul R Burton

**Affiliations:** 1 Data2Knowledge (D2K) Research Group, School of Social and Community Medicine, University of Bristol, Bristol, UK; 2 Centre for Policy, Ethics and Life Sciences (PEALS), Newcastle University, Newcastle, UK; 3 Department of Sociology, University of Cambridge, Cambridge, UK; 4 Urban Cow Productions, London, UK

**Keywords:** ECOUTER, Engagement, ELSI, Empirical ethics, Epistemic, Ontological, New technologies, Mindmap, Translation

## Abstract

**Background:**

Because no single person or group holds knowledge about all aspects of research, mechanisms are needed to support knowledge exchange and engagement. Expertise in the research setting necessarily includes scientific and methodological expertise, but also expertise gained through the experience of participating in research and/or being a recipient of research outcomes (as a patient or member of the public). Engagement is, by its nature, reciprocal and relational: the process of engaging research participants, patients, citizens and others (the many ‘publics’ of engagement) brings them closer to the research but also brings the research closer to them. When translating research into practice, engaging the public and other stakeholders is explicitly intended to make the outcomes of translation relevant to its constituency of users.

**Methods:**

In practice, engagement faces numerous challenges and is often time-consuming, expensive and ‘thorny’ work. We explore the epistemic and ontological considerations and implications of four common critiques of engagement methodologies that contest: representativeness, communication and articulation, impacts and outcome, and democracy. The ECOUTER (Employing COnceptUal schema for policy and Translation Engagement in Research) methodology addresses problems of representation and epistemic foundationalism using a methodology that asks, “How could it be otherwise?” ECOUTER affords the possibility of engagement where spatial and temporal constraints are present, relying on saturation as a method of ‘keeping open’ the possible considerations that might emerge and including reflexive use of qualitative analytic methods.

**Results:**

This paper describes the ECOUTER process, focusing on one worked example and detailing lessons learned from four other pilots. ECOUTER uses mind-mapping techniques to ‘open up’ engagement, iteratively and organically. ECOUTER aims to balance the breadth, accessibility and user-determination of the scope of engagement. An ECOUTER exercise comprises four stages: (1) engagement and knowledge exchange; (2) analysis of mindmap contributions; (3) development of a conceptual schema (i.e. a map of concepts and their relationship); and (4) feedback, refinement and development of recommendations.

**Conclusion:**

ECOUTER refuses fixed truths but also refuses a fixed nature. Its promise lies in its flexibility, adaptability and openness. ECOUTER will be formed and re-formed by the needs and creativity of those who use it.

**Electronic supplementary material:**

The online version of this article (doi:10.1186/s12910-017-0167-z) contains supplementary material, which is available to authorized users.

## Background

### Translational research, stakeholder engagement and genomics

In describing an “ethos and ethics of translation”, Maienschein and colleagues [1] position the rise of translational research in terms of historical transformations in the social contract regarding publicly funded research; specifically, a shift from one in which public investment in science produces public benefit throughout the scientific enterprise *as a whole*, to one in which funding is tied to demonstrable potential for outcomes and where the public (reasonably) expect *scientists* to deliver results. In this newer context they ask us to consider, “*Who decides what count as results*? *And who decides which science will best get us the results desired*? *Who decides what to translate*, *how to do the translation*, *and when something counts as having been successfully translated*? *And on what basis* (*justifiable or not*) *are any of these decisions made*?” [p. 46]. In opening up the question of “*Who decides*?”, they point also to the overwhelming shift in contemporary science and society regarding expectations about who are the relevant stakeholders of today’s science. In a similar way, Callard and colleagues [2] prompt us to ask social, ethical and political questions about the outputs of translational research: Are they available? Are they needed? Do they fit peoples’ lives? In their user/patient-centric model of translational research the authors point us to the identity-producing effects of translational research, asking us to heed Singh and Rose [3] (among others) in involving all those affected by the outputs of research *before* its translation into practice. Engaging the many ‘publics’ who have a stake in decisions about translational outcomes offers one way of ensuring Callard’s questions are aired.

Because no single person or group holds knowledge about all aspects of research, mechanisms are needed to support knowledge exchange and engagement. Moreover, as Burgess [4] notes, there has been a distinctive shift in thinking about governance of bioscience and biotechnology from one in which publics are viewed as needing education about science to one which considers public engagement in the co-production of policy and decision making as offering important local knowledge and expertise. Expertise in the research setting necessarily includes scientific and methodological expertise, but also expertise gained through the experience of participating in research and/or being a recipient of research outcomes (as a patient or member of the public). There is, indeed, a long-standing history of engaging publics and other stakeholders in the varieties of ’omics research; a history as long as the Human Genome project itself. Some of these are bottom-up, led by varying publics, and across boundaries with citizen-science; others comprise what might be called engagement by invitation [5, 6]. It is this latter strategy that is the focus of this paper. And there are many examples of good practice, particularly using deliberative approaches to bring together members of the public with experts in the relevant field [4, 7–23]. While there are many ways to implement deliberative processes [9], in genomics they have typically involved small numbers of participants (up to 25) selected to broadly represent the make-up of the (local) general population; they take place over one or more intensive periods of time; include provision of background material to help participants think through a range of matters and perspectives related to the issues under discussion and the opportunity to interact with and question experts. The output of deliberative engagement is generally the production of recommendations on the issue under discussion.

In ethical terms, exercises in engagement are variously understood as a form of democracy, an act of respect, and an acknowledgement of human rights, including the right to self-determination and the right to be involved in decisions that affect one’s life and life-world [24–30]. The aspiration of such engagement is to improve the alignment of research, healthcare and governmental practices more generally with societal values, and to improve delivery and relevance of services and research outputs or translation. Engagement is, by its nature, reciprocal and relational: the process of engaging research participants, patients, citizens and others (what might be considered the many ‘publics’ of engagement) brings them closer to the research but also brings the research closer to them. In the case of translating research into practice, the focus of this special issue, engaging the public and other stakeholders is explicitly intended to make the outcomes of translation relevant to its constituency of users [31].

### Epistemic and ontological considerations in engagement

In practice, engagement faces a number of challenges. Achieving the aspirations of engagement is time-consuming, expensive and often ‘thorny’ work. While multiple mechanisms for engagement already exist along the spectrum from consultation to control, described originally by Arnstein in 1969 [32], each has its shortcomings and constraints. Some of the most well described engagement mechanisms such as deliberative democracy, deliberative forums, community meetings, consultations, surveys and focus groups (all designed to contribute to decision making) derive both rich and valuable understandings and produce concrete consensual outcomes. However, they can also be costly, time consuming to implement and are available only to participants with the time and capacity to contribute many hours or days to the engagement process, or even more if significant travel from remote communities is required. The commitment of time, energy and presence required of many engagement efforts is difficult, if not impossible, for many individuals. Following Irwin [33], we ask do the ethical and practical aims of engagement hold only if such undertakings are done perfectly? If engagement is tokenistic-if engagement practices reinforce social inequalities by including only those who already have more powerful voices or are disempowering because practices are *merely* instrumental-can the expected outcomes be delivered or deliverable? [34] Moreover, ‘*if there can be no perfect engagement methodology should we abandon the project*?’ To examine more closely the purported shortcomings and constraints of engagement we turn to the typology offered by Irwin and colleagues [33], who identify what amounts to a ‘sport’ of engagement – critique with descriptions running along fairly well trodden lines: *contesting representativeness*, *contesting communication and articulation*, *contesting impacts and outcome*, and *contesting democracy*. In this paper we take to heart Irwin and colleagues’ argument that “*the* (*often implicit*) *evocation of the highest principles that engagement might ideally fulfil can make it difficult to acknowledge and pay serious attention to the varieties of engagement that are very much less than perfect but still somehow* ‘*good*’ [p. 120]”. First, we use their typology as a framework to examine (some) epistemic and ontological rudiments of engagement and their implications for engagement practice.

Epistemically, the critiques identified by Irwin and colleagues are based on a set of foundational assumptions. The first of these, “*Contesting representativeness*” suggests that notions of representation rest on foundational assumptions which hold that representation of large populations, or even of discrete communities, is inherently desirable *and* epistemically or methodologically possible. Critiques of engagement methods, therefore, often round on the sampling techniques that were used; to question whether the right proportions of the right groups of individuals were included in the engagement exercise. To explore this further we take a short detour into sampling methodology.

From a methodological perspective representation raises two critical questions: (1) Does the inferential process in a particular setting actually require representativeness? (2) Even if it is inferentially desirable can representativeness, in practice, be achieved? The first question arises regularly both in quantitative bioscience research and in qualitative research. In the public health setting, for example, if you wish to estimate the prevalence of type-2 diabetes in a given population in order to determine the resources required to maintain a viable service for that population, then the representativeness of the sample – relative to the target population – is crucial to extrapolating the estimate obtained in the sample to generate the implied prevalence in the target population. Here, the required inference is quantitative and could be obtained by integrating the heterogeneous prevalence of type-2 diabetes across many population subgroups carefully weighting the integration for how common each population-subgroup may be. However, the relevant population-subgroups are often unobserved – or unobservable-and the inferred prevalence in the target population then relies on the implicit weights reflected in the *unknown* distribution of subgroups in the sample and in the target population. If those are different, then extrapolation of the sample estimate to the general population may be flawed – potentially badly so. In contrast, in contemporary bioscience, for example in designing major biobanks, the framing of the primary scientific question to be addressed often takes the form: *is there any meaningful association between the* observed *incidence of a disease in a study* (e.g. *new cases of type*-*2 diabetes*) *and the* observed *distribution of a determinant* (*maybe a variant V of gene G*) *in that same study*? Crucially, the estimated association *in the sample* makes no assumption of representativeness-it is simply the ‘observed association in the sample’. In that setting, representativeness only becomes of potential relevance if we try to relate this answer to what it might mean at a general population level. Furthermore, a problem will only arise if the magnitude of the association itself varies markedly between population subgroups: e.g. if variant V of gene G exhibits a strong positive association with the disease in some population subgroups, little or no association in others, and maybe even a negative association in yet others. Although this is scientifically possible, it is likely that the *heterogeneity* between population subgroups of such an association will be less marked than *variation in the prevalence of a disease* between population subgroups. Although it is theoretically possible for a genetic variant that causes a disease in one population subgroup to protect against that same disease in another subgroup, such eventualities are rare. In consequence, provided a sample is of adequate size, it is unlikely that the weak effect of ‘non-representativeness’ alone could convert a null or negative association in the general population to a clearly positive association in the sample or vice versa. This has an important corollary: when representativeness is *not* critical, deliberately designing a study so as to ensure that a sample *is* representative may be scientifically counter-productive if the same resources could instead be put to creating a less representative sample which is more efficient (e.g. deliberately oversampling high-risk population subgroups). The fundamental message is not that ‘representativeness’ is irrelevant but rather that it is sometimes *very important* and sometimes *of little or no relevance*-its importance is dictated entirely by the context of the research question to be asked.

Expectations that engagement should be representative are based on a logic that individuals are or can be representative of the population or community in which they reside or with which they identify. Addressing the second question above, methodologically, it is probably not feasible to *ever* gather such a representative group. In practical and epistemic terms we can never know whether the difference between the (randomly) selected sample is *so different* to the population as to make inferences flawed; i.e. to formally demonstrate that ‘representativeness’ *is* important. This is in part because, in keeping with the logical underpinning of the need for sample representativeness in prevalence studies, the key criteria defining the relevant subgroups comprising a sample to be used for engagement will often be ‘unobserved’ and may be ‘unobservable’. Potential participants who are least likely to be involved in such activities because of temporal, spatial, socio-economic, psychological or emotional factors will likely remain uninvolved if social, structural or other impediments remain unchanged, despite the best efforts to over-sample or otherwise entice them into the process. Recognising the difficulty of circumventing this problem, the pragmatic alternative for recruitment in engagement is to accept the impossibility of attaining and demonstrating ‘representativeness’ and instead to pursue the benefits provided by saturation. Saturation is a term most often used in the context of qualitative research where data collection (interviews, ethnographic observations, interactions, documents) ceases at some point after which no new themes, concepts, theoretical components or other phenomena emerge from the data. Rather than assume an infinite variety of possible individuals and the implied boundlessness of perspectives, saturation relies upon the rather more limited variety of difference (or différance [35]), discourse [36], social repertoires [37] and other markers of a socially-constituted world that might be accessible through the input of socially-situated individuals. To take this seriously in engagement would mean to strive to recognise difference, diversity and alterity – it is to continue to ask the question ‘How could this be otherwise?’ [38] until no new alternatives emerge. Where ‘this’ is the phenomenon under consideration and ‘continuation’ is enacted by reaching out to embrace difference. In practical terms saturation offers an alternative to random (or quasi-random) selection of engagement participants for representativeness. Rather than selecting on the basis of a supposed ‘statistically representative’ sample, purposive selection of potential participants until saturation is reached may offer at least some access to the alterity in a heterogeneous population. Crucially, as a direct analogue to the quantitative setting (above), this is both valid and useful in describing the complex interrelationships between ideas, understanding and viewpoints *in the engagement sample* and making the valid claim that these findings represent a useful snapshot of the relationships that exist in the broader population. Inferential problems only arise if attempts are then made to make precise *quantitative* statements about the frequency with which particular ideas might occur in the broader population or about the strength of the association between different ideas. The basis of purposive selection and the determination of saturation will necessarily be engagement specific. And this brings us to a second, related, foundational assumption.

Notions of representation implicitly defend foundational assumptions of truth: that there is or might be knowable, genuine, often ‘lay’ perspectives that we can gain access to if only we use the right method or approach. As Irwin et al. [33] suggest, *contesting communication method and articulation* takes the form of questions about what they call the “conditions of speech”: how and whether participants in an engagement exercise are able to articulate their views in “a proper and meaningful manner”, including whether any material presented to them is sufficiently balanced or unbiased, too superficial or too complex, or framed to produce certain outcomes. Central to such critiques is the notion that there is an ideal, neutral or ‘objective’ set of truths which can be articulated. Certainly, to actively bias material or encourage extremist perspectives would be unacceptable. But claims to objective truth are intrinsically problematic. Different notions of evidence and expertise make claims to different values (e.g. patient or participant centredness) and position particular forms of evidence (e.g. quantitative research data) as particularly real, true or authoritative. Favouring one notion of evidence and expertise over others narrows the epistemic landscape by side-lining incompatible or contradictory notions. The purpose in seeking ‘public’ views is often to counter dominant views about a particular phenomenon, especially those which may reproduce social inequalities or other disadvantage. In the dialectic of powerful/less voices and perspectives, the scientist is contrasted to research participant, doctor to patient, government to citizen. Addressing power relations is politically important work but if a commitment to verity simply results in truth-contests this work may be counter-productive; if, for example, privileged access to ‘truth’ is seen to be the province of one set of actors alone.

Attempts to achieve consensus, such as in deliberative forms of engagement, aim to avert the potential stalemate of competing truths. But the implicit focus on consensus, even by contesting it, may potentially miss a key component of the “conditions of speech”. Language (following Austin, Foucault, Butler and others) is active. Language is not merely representational, expressing well or poorly some underlying truth or giving access (or not) to some alternative perspective [39]. When we speak, we accomplish a range of actions. The perspectives we offer and the values or discourses we draw upon bring into being certain versions of the world. They construct or enact the social, but they do more than that. The ‘truths’ so constructed lay out the boundaries of the possible. A world in which multiple publics voices are heard is fundamentally different to one in which those same voices are absent or suppressed. While public voices may still be marginalised, the very possibility of engagement can arguably act as a form of the conduct of conduct [40, 41]: scientists, doctors and governments police their own practices on the understanding that those practices may be scrutinised.

The active character of language is not restricted to enactment but also of situated action; that is, bringing about certain actions in relationship. As individuals, we produce and co-produce our ‘selves’ as well as our worlds. We present ourselves – or in Butlerian terms [42, 43] ‘perform’ ourselves – as certain types of people (e.g. in terms of gender, ethnicity, expertise). We act to ‘save face’ [44, 45], especially in interactions which may challenge our ‘selves’, and we warrant some actions or outcomes over others. Understanding language as performative leads us to two particular considerations in terms of engagement. First, that representation itself (in the form of participants’ views or perspectives within engagement practices) cannot be taken simply or straightforwardly as depictions of truth or fact. This is not to say that these representations are untrue or not firmly held as beliefs, but rather that they construct certain world views, often with particular value commitments. *All* contributions, whether by participants or organisers, in an exercise of engagement are produced from specific subject positions which may change in different settings or be deployed for different effects; this is as true of the most senior collaborator as of those less powerfully positioned. It is these constructs (of world and self) that are so enormously powerful analytically, but which are often overlooked in engagement practice. By establishing certain truths or values as normative (what ‘should be’), the implications of what *can be*, what is made possible in practice within the provisions of these truths or values are also laid out, These are undoubtedly worthy of analytic attention. The second consideration is that these world views and value commitments themselves implicate certain actions or outcomes. The critiques identified by Irwin et al. [33] as *contested impact and outcomes* are undoubtedly recognisable in the oft voiced claim that a particular engagement exercise has had no impact. While it may be true (a material reality) that none of the recommendations of a particular engagement exercise have been implemented, it is unlikely that there has been *no* effect, even if this is in ways that are unexpected or difficult to discern. Engagement practitioners do well to look also to these unintended effects as evidence of influence and to actively follow the possibilities and opportunities they present. Though not all unintended outcomes will be as wished, some just might be: take for example, the deliberative community engagement exercise undertaken prior to the establishment of the Mayo Clinic Biobank which resulted in the establishment of an ongoing Community Advisory Board to provide advice, review policy and participant materials, and provide input on complex policy issues [22].

These ontological considerations of engagement practice and its impact bring us to the final strand of the common challenge described by Irwin and colleagues: *contesting democracy*. The question ‘What is democracy?’ can certainly be considered an epistemic issue – forests have been felled in presenting potential answers – and in the same way that foundational concepts of truth are epistemically constraining, so too are concepts of democracy. But democracy is also a profoundly ontological issue: it is something we *do*. Therefore rather than contesting definitions of democracy or the opportunity cost of not picking the ‘right’ one, the authors take the position that the enactment of something *called* democracy in achieving the pragmatic aspirations of engagement, in and with translational research, is rather less important than *doing* something that might or might not achieve such lofty ideals.

To return to the question of this section ‘if there can be no perfect engagement methodology should we abandon the project?’ We suggest not. It is impossible (and perhaps folly to try) to conceive of a single mechanism that would suit all (potential) engagement settings, purposes and communities. Instead, we offer here an approach that attempts to address problems of representation and epistemic foundationalism using a newly developed methodology that seeks to maintain focus on the question, ‘How could it be otherwise?’ We embrace alterity by offering an approach that we believe affords the possibility of engagement where spatial and temporal constraints are present, and which relies on saturation as a method of ‘keeping open’ the possible considerations that might emerge from that engagement. We also address the ontological challenges by introducing an analytic element to the engagement process. However, as will become evident, the method we describe here is not an alternative that should be viewed as addressing all concerns about existing approaches to engagement. Rather it is an approach that may provide a useful complement to existing methods and indeed borrows from some of those methods in its realisation. We are intentionally catholic in our embracing of multiple engagement and analytic methodologies.

## Methods

### The ECOUTER engagement method

The ECOUTER (Employing COnceptUal schema for policy and Translation Engagement in Research) [46] methodology is our response to the epistemic and ontological considerations discussed above. ECOUTER offers an alternative ontology for and of engagement without claiming to solve *all* of the challenges of engagement. It is instead anticipated that ECOUTER will complement and work in combination with other existing approaches. Taken from the French verb ‘to listen’, ECOUTER is an engagement approach that uses interactive mind-mapping – in low or high-tech formats – to enable stakeholders to draw upon and explore their own knowledge (we do not assume individual knowledge is static or complete), to consider other relevant knowledge and to interact on topics of shared concern. ECOUTER recognises that many forms of expertise must be drawn upon to ensure that decision making about research policy, governance, priorities and practice is robust, timely and appropriate; further, it recognises that decisions made about publicly funded research must be aligned with social needs and values in order to realise their optimal translation into societal benefit. ECOUTER does not assume that all contributions made in an engagement exercise are themselves (or become via the engagement process) *prima facie* evidence. Instead, participant contributions, or first order constructs (participant produced), are subject to qualitative forms of analysis so as to derive second order constructs (researcher produced) that then form a conceptual model and related recommendations. These are then fed back to stakeholders for further refinement.

In its online form, ECOUTER incorporates knowledge exchange by enabling online access to external information sources. While many other engagement mechanisms support engagement they can also constrain the range of possible understandings by using pre-determined categories or may intentionally or inadvertently circumscribe discussion through the particular framework used or assumptions imposed. ECOUTER uses mind-mapping techniques to ‘open up’ engagement, iteratively and organically. It explicitly supports the inductive identification and exploration of new ideas or topics. Although all approaches to engagement sacrifice some utility or depth, ECOUTER aims to find a balance between breadth, accessibility and user-determination of the scope of engagement.

In practice, an ECOUTER exercise comprises four stages: Stage 1 – engagement and knowledge exchange; Stage 2 – analysis of mindmap contributions; Stage 3 – development of a conceptual schema (i.e. a map of concepts and their relationships); and Stage 4 – iterative feedback, refinement and development of recommendations where appropriate. Once completed, the mindmap is analysed iteratively using established qualitative techniques (e.g. thematic analysis or discourse analysis). The ECOUTER analysis is not dependent on the means of data collection. A conceptual schema, a map of concepts and their relationships, is developed collaboratively. The results are further discussed with the participants or, where participation is fleeting and anonymous, with participants from similar stakeholder communities. Finally, the conceptual schema(s) and feedback iterations form the basis of recommendations for research, governance, practice and/or policy. In this paper we describe the first three stages of the ECOUTER process, primarily using the experience of the ‘HeLEX’ ECOUTER (E3, one of five ECOUTER pilots, E1-E5, described below) as a worked example. The final part of an ECOUTER has not yet been undertaken for any of the pilots and therefore is not addressed in detail here.

The ‘HeLEX’ ECOUTER (E3) was conducted during an academic conference in June 2015 (E3) and asked “*Translation and emerging technologies*: *what are the social*, *ethical and legal issues*?” Hosted by HeLEX (helex@dph.ox.ac.uk) and led by the ELSI2.0 Collaboratory (https://elsi2workspace.tghn.org/), a community of scholars interested in the Ethical, Legal and Social Implications (ELSI) of genetics and genomics, the stated aim of *Translation in Healthcare* conference, June 2015, was to “[*bring*] *together a wide range of voices to discuss and think more deeply about the technological*, *legal*, *ethical*, *and social challenges raised by new technologies in healthcare* … *to capture the energy and free flow of ideas that usually only occurs in the coffee breaks of most conferences*”^1^. ECOUTER was one of the methods used in the conference to facilitate discussion and gather the range of perspectives of delegates.

## Results

### Stage 1: Engagement and knowledge exchange

ECOUTER offers face-to-face and online modes of engagement to support participation by a broad range of people. An ECOUTER exercise begins by posing a central question and typically seeds the mindmap with a small number of initial themes/sub-questions as well as links to materials in the relevant evidence base where possible. Participants then draw upon their own knowledge of cognate issues to respond to and contribute ideas to a mindmap, including links to additional evidence (though the process does not assume either type of evidence to be complete). The ECOUTER team records the evolving mindmap data at various stages, monitors for (in) appropriate activity, and alerts participants of end dates. In the online form of an ECOUTER exercise, participants may access the mindmap at a time that suits them and as often as they wish; input is entirely anonymous to both contributors and facilitators of the mindmap. In its face-to-face form, a booth is typically set up in a high traffic area and staffed with ECOUTER facilitators, who interact with participants and help them capture their ideas and thoughts on a mindmap using tablets and laptops or on a wall using Post-It notes.

During the lunch break on day two of the *Translation* conference (E3), an ECOUTER exhibition space was set up in a high traffic location and staffed by 7 members of the D2K research group, including a videographer. The exhibition space was equipped with three laptops providing access to the online mindmapping website as well as two 60” monitors, one displaying a live version of the mindmap as it evolved and the other for delegates to explore the same mindmap in detail on a big screen. Technical details about the establishment of the ECOUTER mindmap, for online and offline modes, are beyond the scope of this paper and are available elsewhere [47, 48].

The mindmap [see Additional file 1: Figure S1: ECOUTER mindmap and Additional file 2: ECOUTER mindmap output] began with a central question reflecting the conference theme and was pre-seeded with six questions developed by MJM and JTM based on key issues raised by speakers in the first plenary session and designed to provoke comment. During the ECOUTER, participants drew on their own understandings and often their own research but were not otherwise asked to link out to external evidence as would normally be the case. Instead, the external evidence base providing the counterpoint for participant reflection consisted of the presentations delivered in plenary on the first day of the conference. Given the setting, numerous “micro-discussions” took place between participants and facilitators. Results from these exchanges were added to the mindmap by D2K members with participants’ permission. Approximately two thirds of 119 conference delegates who were registered on the day stopped by the exhibition stand to discuss the ECOUTER method as well as the questions posed on research translation. A total of 37 entries were made in the mindmap over approximately 75 min, with a small number of participants contributing more than one entry and a large number jointly contributed single entries. The setting made complete anonymity unlikely and facilitators were able to see what some participants wrote. Nonetheless, the map displayed was anonymous.

### Stage 2: Analysis of mindmap contributions

Stage 2 of the ECOUTER involved analysing the first order constructs placed on the mindmap (i.e. those produced by the participants in their contributions to the mindmap). While it might be argued that such constructs should speak for themselves (and indeed the raw mindmap is included as Figure 1 and a list of the contributions as Figure 2), in practice participants’ efforts to summarise their thoughts for inclusion in the mindmap typically reflect an imperfect process which requires some unpacking on the part of those doing the analysis.

A preliminary analysis of the content of the mindmap was presented by MJM in plenary session on the final day of the conference. Following the conference, MJM and JTM each subsequently analysed the mindmap entries thematically before working iteratively until agreement was reached on top level themes and sub-themes using the constant comparative method [49]. This preliminary analysis was then discussed during data analysis sessions (MJM, JTM, AT, MB, CO) where first order constructs (those produced by participants in the mindmap) were interrogated and their meaning clarified. Analysis was supplemented by the micro-discussions participants engaged in with facilitators as the mindmap was being populated. The analysis here is intentionally descriptive and strongly tied to the empirical material. While any broadly thematic or content-based form of analysis could be used in Stage 2, we did not want to move too far from the data itself in order that the next stage of analysis could be directly informed by our explication of the first order constructs. This analysis comprised Stage 2 of ECOUTER exercise and is shown in Additional file 3: *Analysis of participant contributions in an ECOUTER* – *first order constructs*. Stage 2 of the ECOUTER process demonstrated that participants viewed translation as a complex process which included a range of stakeholders who themselves represented a broad range of perspectives and experiences that could contribute to translation.

### Stage 3: Development of a conceptual schema

Stage 3 of the ECOUTER method comprised further analysis of the first order constructs of the participants. We first looked to the construction of certain objects (e.g. translation) and subjects (e.g. patient, scientist): this type of analysis is closest to Foucaultian forms of discourse analysis [39, 50–52]. Taking the position that the values expressed in language are (as claimed of language itself [42, 53–55]) performative, we examined the implications (or effects) of the epistemic and non-epistemic values (i.e. knowledge-related and socio-ethical values, assumptions and standpoints) called upon by conference delegates to describe the social, ethical, political or legal issues for translation in healthcare. In using this particular combination of analysis we are deliberately intending to address the epistemic and ontological foundations we discussed earlier. However, any form of analysis which goes beyond ‘face value’ representation would be workable, though it should preferably be reflexive and identify its own epistemic and ontological commitments. The analysis was used to produce second order constructs (researchers’ constructs) and thereby build the conceptual schema, a ‘map’ of concepts and their relationships. The analysis, described in Additional file 4: *ECOUTER conceptual schema* – *second order constructs*, presents an interrelated trio of concepts we call *Perspectives*, *Processes* and *People*.

When considered in its entirety, the second order conceptual schema demonstrates the ways in which the ECOUTER methodology facilitated engagement during the conference and opened up the subject of translation in healthcare to wider examination beyond what was said by the conference speakers. ECOUTER offered delegates a forum for challenging the perspectives dominant in scholarly frameworks and provided the means to engage the cross section of delegates representing a variety of interests in the field of translation. Moreover, the ECOUTER helped ‘open up’ the discourses embedded in the conference programme by challenging and advancing ideas on how the process of translation happens. In particular, delegates underscored the ways in which emerging healthcare technologies are themselves a highly ‘peopled’ phenomena, that is, one which is profoundly grounded in human actions and relationships. While some of the contributions to the mindmap were also heard in the plenary sessions (both in specific presentations and in general Q&A exchanges), ECOUTER amplified the dialogue among the delegates by offering the opportunity to elaborate and expand beyond the confines of the presentations, especially to junior academics who may have been reluctant to contribute their ideas in plenary and other sessions.

### Stage 4: Feedback, refinement and development of recommendations

As noted above, we have not yet completed a full cycle of the ECOUTER method and therefore do not present a ‘worked’ Stage 4. In practice, Stage 4 could be deployed using a number of existing deliberative or other engagement approaches [7–9, 11, 16, 20, 28, 56–68]. In these cases, the analysis in Stages 2 and 3 would form part of the material upon which to reflect or deliberate.

## Discussion

### Piloting ECOUTER

The ECOUTER methodology has been – or is being – piloted in five settings. Completed pilots have been conducted with: the international ELSI community on the topic of trust in data linkage (E1); the general public on the use of personal medical records in research (E2); delegates of an academic conference (E3), the focus of this paper; and, researchers attending the closing workshop of a multi-year European research project to develop tools and methods facilitating data sharing and biobanks (E4). The final ECOUTER pilot (E5), is currently being used as a tool for engaging with birth cohort participants as they attend whole-day data collection clinics (2015–2017) around the age of 24 years.

Each pilot has contributed to the development of ECOUTER both in terms of delivery and analysis. E1, the ‘P^3^G ECOUTER’, was conducted entirely online over a 5 week period in September/October 2014 under the auspices of P^3^G (Public Population Project in Genomics and Society) at McGill University, Canada. The purpose was to explore innovative mechanisms to build trust in human health research biobanking. The ECOUTER started with the question, *What are the ethical*, *legal and social issues related to trust in data linkage*? Existing ELSI-related distribution lists were used to extend approximately 175 invitations to stakeholders: 58 ‘ELSI stakeholders’ in 11 countries (across Europe, Africa, North and South America) registered to participate. E1 received just over 100 contributions and confirmed the proof of concept for use of ECOUTER to facilitate discussions within a stakeholder community distributed over a large non-contiguous geographic area.

E2, the ‘Shopping Centre ECOUTER’ tested ECOUTER as a tool for public engagement at the local level. The topic was the use of personal health records for research, following the then recently abandoned initial rollout of the care (dot) data initiative in the English NHS. On a single day in November 2014, a booth was set up in a large urban shopping centre in the UK offering members of the public tablet computers and a large screen monitor with which to consider the question, *Your medical records*: *handover or hands off*? Seven facilitators initiated micro-discussions with over 100 members of the public, as a result of which 83 contributions were made to the mind map. Shopping Centre ECOUTER demonstrated the efficacy of the ECOUTER methodology when conducted face-to-face and on a topic involving experiential expertise. Given their close proximity timewise and their similarities of topic, the results of the Shopping Centre and P^3^G ECOUTERs were analysed jointly, revealing a high degree of conceptual overlap. Despite differences in the two originating questions, multiple intersecting themes emerged yielding a conceptual schema comprising four areas: definitions and boundaries; oversight mechanisms; threats; and new knowledge. Nonetheless, it was significant that some issues were emphasised more by the public Shopping Centre ECOUTER participants: concern about confidentiality and anonymity; concern about exploitation for profit; and, support for data used for research. ECOUTER enabled engagement and facilitated the surfacing of diverse points of view from stakeholder communities of differing ‘status’.

E3, the ‘HeLEX’ ECOUTER, through which we explore the methodology in this paper, is described in detailed above.

Technical limitations (i.e. the unknown room layout/availability of power points, etc., plus the logistical difficulties of transporting display equipment to another country) in the fourth pilot (E4) meant this one-day ECOUTER was conducted using Post-It notes to record the discussions. The event was the final meeting of a 5 year European funded consortium, BioSHaRE (www.bioshare.eu/), at which project participants were invited to consider results from a formal evaluation of new data sharing tools produced by the project [69]. Individuals were invited to review a print copy of key findings from the first two stages of the evaluation (interviews and a survey) presented in a mindmap form. They were then asked to consider the question, *BioSHaRE tools*: *Where to now*? Responses were written on Post-It notes and attached to a wall in the lunch/breakout area of the workshop venue. The ‘BioSHaRE ECOUTER’ unfolded primarily during breaks in the meeting as participants gathered for refreshments. Of the 118 people attending the event, we counted 112 actively engaged with the ECOUTER team and a total of 117 Post-It notes were contributed to the map. Analysis of the results indicated broad coherence between the findings and of the evaluation. Participants shared concerns for usability challenges, data access, and the need to address ELSI-related barriers. What was perhaps most surprising was the degree to which contributions to the BioSHaRE ECOUTER emphasised different and often more positive aspects of these issues, particularly the users’ perspective (e.g. need for workshops using own data, tool integration, and user needs assessment) than present in the evaluation which largely focused on the developers and first users of the tools. E4 demonstrated the solution building capacity of ECOUTER as the results led both to the identification of recommendations grounded in participant’s experiences and to the development of new ideas and approaches for subsequent grant applications.

E5 is an ECOUTER underway since May 2016 with participants in the UK Children of the 90s study, the Avon Longitudinal Study of Parents and Children (ALSPAC), in its 24+ data collection clinic (http://www.bristol.ac.uk/alspac/). ‘Clinic ECOUTER’ is part of ALSPAC’s commitment to participant engagement, which has included, from 2006, a participant advisory group called the Teenage Advisory Panel (TAP) and, since 2013, the Original Cohort Advisory Panel [70]. Since the study’s inception a key governance and policy group, the ALSPAC Law and Ethics Committee (ALEC), has included study participants with two parent members and, from its establishment, two TAP representatives. ALEC is now formally constituted to include equal numbers of participant and non-participant members and is currently chaired by by a study participant. Clinic ECOUTER asks participants attending ‘*What areas would you like Children of the 90s to research*?’ The data collection clinic occurs over a 24 month period with ECOUTER running mid-clinic. The long duration offers challenges for engagement which ECOUTER is uniquely placed to address (the temporal distance of participants) but also offers the possibility of comparing different ECOUTER modes. Participants are encouraged to interact with a mindmap during breaks in their visits but later in the data collection period, participants will also be offered online access to the mindmap outside of the clinic. Results will be analysed throughout the contribution period meaning that the ECOUTER will be iterative among the participants as they cycle through clinic attendance and beyond. Changes in interactions produced by different modes will be analysed to improve ECOUTER.

### Doing ECOUTER

Beyond the ‘worked’ example presented here, information on how to conduct an ECOUTER can be found in two locations. Technical aspects of running an event, including discussion of ongoing challenges such as the limitations of using open source software, have been published online [48], while a Wiki to support facilitators with checklists and other practical documentation is available online and updated regularly. Beyond this, facilitators have identified a number of concerns and limitations from the five pilot events discussed in this paper. First, both online and face-to-face ECOUTERs require a high degree of regular moderation in order to keep discussions active and engaging. This is particularly true with ECOUTERs involving interactive booths, where facilitators must find ways to transfer the content of micro-discussions to the mind map. Second, there is an ongoing issue of identifying how many individuals eventually contribute to an ECOUTER mind map. Equally, it can be difficult at busy times to count how many individuals visit a booth versus how many then go online. Accurate figures for both total number of participants and which contributions were made by whom will ultimately be required as part of evaluating the exercise. Third, there remain ethical aspects yet to be fully addressed, including how best to inform participants in advance in face-to-face settings. Especially in the exhibition setting (e.g. E2, E3 and E4) interactions with facilitators can be somewhat brief; better systems are needed to inform individuals about the process without being overly time consuming. Finally, the ECOUTER method has not yet completed its intended final cycle of feedback to participants. While aspects of this were achieved in the BioSHaRE ECOUTER (E4), the intention is to have participants (or a similar stakeholder community) review more fully the conceptual schema arising from analysis: we are currently planning Stage 4 for E1/2.

## Conclusion

### Where does ECOUTER ‘fit’ as an engagement strategy?

Finally, having outlined a range of epistemic and ontological challenges to engagement and having presented one particular methodology in response, what have we produced? We have taken Irwin and colleague’s typology as an armature, though we doubt if it was ever intended thus, to build the logics of ECOUTER. Against representativeness we offer saturation as a way of surfacing diverse ideas and discourses; rather than discovery of static (or ‘underlying’) truths - views, perspectives, voices; we suggest using mindmapping a means of ‘opening up’ exploration; we consider the contributions and interactions enacted in those mindmaps as material for analysis; looking to the performative nature of language we propose looking to ‘how things could be otherwise’, not how things *should* be (for who might be the arbiter of such ‘shoulds’?) but how things *can* be; and, moreover, how ‘democracy’ itself could be otherwise. And, *a la* Spranzi and Brunet [23], what is interesting about participants’ contributions is the *values* they emphasise: while not the focus of analysis in this paper, values will be central to our consideration of trust and data linkage (E1 and 2). In practical terms ECOUTER offers what we hope is an affordable methodology which is flexible and accessible in a range of settings. What our pilots demonstrate, beyond how much is still to be learned, is that the method can elicit a range of new ideas and new possibilities and that these can and do travel beyond the dominant or prevailing discourses. But this is only achieved by using qualitative analytic methods reflexively. So, is this engagement or is this research? ECOUTER is clearly not deliberative democracy: though as we have noted Stage 4 may borrow from those methods. And yet it is, as per Scott and colleagues description of deliberative democracy [24], “more than simply summing up or aggregating opinions in some co-ordinated fashion” (p.4). Does it, then, sit within definitions [71, 72] of empirical ethics? Perhaps, but not easily. To take Davies and colleagues useful typology [72] it is neither purely dialogical nor purely consultative; it is hybrid and, we suggest, perpetually so. In contrast to the other hybrid forms of empirical bioethics described by Davies et al., ECOUTER is founded on an antifoundationalist epistemology and ontology, following Foucault [36, 40, 41, 73], Bulter [43] and Woolgar [38], particularly it is concerned with the situated [74] and relational [75] ethics. Or perhaps, where a normative stance is deployed analytically, rhetorically, pragmatically or politically, it may remain, as Ives’ quasi-foundational ‘reflexive balancing’ [76], open to revision, challenge and reassessment. Refusing fixed truths, ECOUTER also refuses a fixed nature. In order that it achieve its promise it must remain flexible, adaptable and open. ECOUTER will be formed and re-formed by the needs and creativity of those who use it.

## Additional files

Additional file 1: Figure S1. ECOUTER mindmap. Image of final mindmap created during conference ECOUTER event (DOCX 226 kb)

Additional file 2: ECOUTER mindmap output. Contributions to ECOUTER mindmap in text form (DOCX 14 kb)

Additional file 3: Analysis of participant contributions in an ECOUTER-first order constructs. Output of first order data analysis (DOCX 17 kb)

Additional file 4: ECOUTER conceptual schema-second order constructs. Description of data: Output of second order data analysis (DOCX 13 kb)

## Footnotes

1 A programme of the conference is available at http://www.ndph.ox.ac.uk/upcoming-events/translation-in-healthcare/conference-programme-2015-v53-with-sponsors-for-website.pdf.

## References

[1] Maienschein J (2008). The ethos and ethics of translational research. Am J Bioeth.

[2] Callard F, Rose D, Wykes T (2012). Close to the bench as well as at the bedside: involving service users in all phases of translational research. Health Expect.

[3] Singh I, Rose N (2009). Biomarkers in psychiatry. Nat.

[4] Burgess MM (2014). From ‘trust us’ to participatory governance: deliberative publics and science policy. Public Underst Sci.

[5] Wynne B (2007). Public participation in science and technology: performing and obscuring a political–conceptual category mistake. East Asian Sci Technol Soc.

[6] Kaye J (2012). From patients to partners: participant-centric initiatives in biomedical research. Nat Rev Genet.

[7] Secko DM, Burgess M, O’Doherty K (2008). Perspectives on engaging the public in the ethics of emerging biotechnologies: from salmon to biobanks to neuroethics. Account Res.

[8] Garrett SB, Dohan D, Koenig BA (2015). Linking broad consent to biobank governance: support from a deliberative public engagement in California. Am J Bioeth.

[9] O’doherty K (2012). Implementing a public deliberative forum. Hastings Cent Rep.

[10] McWhirter RE (2014). Community engagement for big epidemiology: deliberative democracy as a tool. J Pers Med.

[11] Lemke AA (2010). Community engagement in biobanking: experiences from the eMERGE network. Genomics Soc Policy.

[12] O’Doherty KC, Hawkins A (2010). Structuring public engagement for effective input in policy development on human tissue biobanking. Public Health Genomics.

[13] Watt AM (2012). The ASTUTE health study protocol: deliberative stakeholder engagements to inform implementation approaches to healthcare disinvestment. Implement Sci.

[14] Secko DM (2009). Informed consent in biobank research: a deliberative approach to the debate. Soc Sci Med.

[15] O'Doherty K (2012). Managing the introduction of biobanks to potential participants: lessons from a deliberative public forum. Biopreserv Biobank.

[16] O'Doherty K, Burgess M, Secko DM (2010). Sequencing the salmon genome: a deliberative public engagement. Life Sci Soc Policy.

[17] Molster C (2013). I*nforming public health policy through deliberative public engagement*: *perceived impact on participants and citizen*–*government relations*. Genet Test Mol Biomarkers.

[18] Mackenzie MK, Warren ME (2012). *Two trust*-*based uses of minipublics in democratic systems*. Deliberative systems: deliberative democracy at the large scale.

[19] Burgess MM. *13 Deriving policy and governance from deliberative events and mini-publics.* Regulating Next Generation Agri-Food Biotechnologies: Lessons from European, North American and Asian Experiences. 2013;220.

[20] Burgess MM, O’Doherty K. *Deliberative public engagement related to governing biobanks: final report.* 2007. http://tinyurl.com/h9s2mjq. Accessed 14 Nov 2016

[21] Garrett SB (2015). EngageUC: developing an efficient and ethical approach to biobanking research at the University of California. Clin Transl Sci.

[22] Olson JE (2013). The Mayo Clinic biobank: a building block for individualized medicine. Mayo Clin Proc.

[23] Spranzi M, Brunet L (2015). The French bioethics public consultation and the anonymity doctrine: empirical ethics and normative assumptions. Monash Bioeth Rev.

[24] Kim SY (2009). Assessing the public’s views in research ethics controversies: deliberative democracy and bioethics as natural allies. J Empir Res Hum Res Ethics.

[25] Gottweis H, Gaskell G, Starkbaum J (2011). Connecting the public with biobank research: reciprocity matters. Nat Rev Genet.

[26] 27. Winickoff DE. *From benefit sharing to power sharing: partnership governance in population genomics research.* In: Center for the Study of Law and Society Jurisprudence and Social Policy Program. 2008. https://escholarship.org/uc/item/845393hh. Accessed 14 Nov 2016

[27] Stirling A. P*ower, truth and progress: towards knowledge democracies in Europe.* In: Future directions for scientific advice in Europe. 2015. http://www.csap.cam.ac.uk/media/uploads/files/1/future-directions-for-scientific-advice-in-europe-v6a-online.pdf#page=133. Accessed 14 Nov 2016

[28] Solberg B (2015). Biobank consent models–are we moving toward increased participant engagement in biobanking?. J Biorepos Sci Appl Med.

[29] Rial-Sebbag E, Cambon-Thomsen A, Mascalzone D (2015). Governing biobanks through a European infrastructure. Ethics, law and governance of biobanking: national, European and international approaches.

[30] O’Doherty K, Einsiedel E (2012). Public engagement and emerging technologies.

[31] Murtagh MJ (2011). Realizing the promise of population biobanks: a new model for translation. Hum Genet.

[32] Arnstein SR (1969). A ladder of citizen participation. J Am Inst Plann.

[33] Irwin A, Jensen TE, Jones KE (2013). The good, the bad and the perfect: criticizing engagement practice. Soc Stud Sci..

[34] Thomson R, Murtagh M, Khaw F (2005). Tensions in public health policy: patient engagement, evidence-based public health and health inequalities. Qual Saf Health Care.

[35] Derrida J (2016). Of grammatology.

[36] Foucault M (2015). The archaeology of knowledge.

[37] Potter J, Wetherell M (1987). Discourse and social psychology: beyond attitudes and behaviour.

[38] Woolgar S, Lezaun J (2013). The wrong bin bag: a turn to ontology in science and technology studies?. Soc Stud Sci.

[39] Potter J (1996). Representing reality: discourse, rhetoric and social construction.

[40] Foucault M (2008). The birth of biopolitics: lectures at the Collège de France, 1978–1979.

[41] Foucault M (1982). The subject and power. Crit Inq.

[42] Butler J (1990). Gender trouble and the subversion of identity.

[43] Butler J (1997). Excitable speech: a politics of the performative.

[44] Goffman E (1955). On face-work: an analysis of ritual elements in social interaction. Psychiatry.

[45] Goffman, E. On face-work. In Interaction Ritual: Essays on Face-to-Face Ritual. Harmondsworth UK, 1967:5–45

[46] University of Bristol: ECOUTER. http://tinyurl.com/hsp8p8c (2016). Accessed 14 Nov 2016

[47] Wilson RC. *ECOUTER wiki space*. https://wikis.bris.ac.uk/display/ECOUT (2016). Accessed 14 Nov 2016

[48] Wilson RC, et al. *Digital methodology to implement the ECOUTER engagement process.* F1000Res. 2016; doi: 10.12688/f1000research.8786.1. 10.12688/f1000research.8786.1 PMC4911626 27366320

[49] Glaser BG, Strauss AL (1965). The constant comparative method of qualitative analysis. Soc Probl.

[50] Antaki C, et al. *Discourse analysis means doing analysis: A critique of six analytic shortcomings.* 2003. https://dspace.lboro.ac.uk/2134/633. Accessed 14 Nov 2016.

[51] Diaz-Bone R (2008). The field of Foucaultian discourse analysis: Structures, developments and perspectives. Hist Soz Forsch.

[52] Hacking I (2004). Between Michel Foucault and Erving Goffman: between discourse in the abstract and face-to-face interaction. Econ Soc.

[53] Austin JL (1975). How to do things with words.

[54] Searle JR (1969). Speech acts: an essay in the philosophy of language.

[55] Foucault M (1970). The order of things: an archaeology of the human sciences.

[56] Abelson J (2013). Public deliberation in health policy and bioethics: mapping an emerging, interdisciplinary field. J Public Deliberation.

[57] Avard D (2009). Public health genomics (PHG) and public participation: points to consider. J Public Deliberation.

[58] Chalmers D (2014). New avenues within community engagement: addressing the ingenuity gap in our approach to health research and future provision of health care. J Responsible Innov.

[59] Cohn EG (2015). Increasing participation in genomic research and biobanking through community-based capacity building. J Genet Couns.

[60] Etchegary H (2013). Consulting the community: public expectations and attitudes about genetics research. Eur J Hum Genet.

[61] Godard B, Marshall J, Laberge C (2007). Community engagement in genetic research: results of the first public consultation for the Quebec CARTaGENE project. Public Health Genomics.

[62] Lemke AA, Harris-Wai JN (2015). Stakeholder engagement in policy development: challenges and opportunities for human genomics. Genet Med.

[63] O’Doherty KC, Burgess MM (2008). Engaging the public on biobanks: outcomes of the BC biobank deliberation. Public Health Genomics.

[64] O’Doherty KC, Burgess MM (2013). Public deliberation to develop ethical norms and inform policy for biobanks: lessons learnt and challenges remaining. Res Ethics.

[65] Peacock SJ. *Public attitudes and values in priority setting.* Isr J Health Policy Res. 2015. doi: 10.1186/s13584-015-0025-8. 10.1186/s13584-015-0025-8 PMC4474577 26097679

[66] Rikkers W, et al. *Two methods for engaging with the community in setting priorities for child health research: who engages?* PloS One. 2015. doi:10.1371/journal.pone.0125969. 10.1371/journal.pone.0125969 PMC4418596 25938240

[67] Tello-Rozas S, Pozzebon M, Mailhot C (2015). Uncovering micro-practices and pathways of engagement that scale up social-driven collaborations: a practice view of power. J Manage Stud.

[68] Wilcox ES (2008). A lego model to help inform participants at the British Columbia Biobank deliberation. Health Law Rev.

[69] Murtagh MJ (2016). International data sharing in practice: new technologies meet old governance. Biopreserv Biobank.

[70] Boyd A (2013). Cohort profile: the ‘Children of the 90s’ - the index offspring of the Avon Longitudinal Study of Parents and Children. Int J Epidemiol.

[71] Molewijk AC (2003). Implicit normativity in evidence-based medicine: a plea for integrated empirical ethics research. Health Care Anal.

[72] Davies R, Ives J, Dunn M. *A systematic review of empirical bioethics methodologies.* BMC Med Ethics. 2015. doi:10.1186/s12910-015-0010-3. 10.1186/s12910-015-0010-3 PMC4357052 25885575

[73] Burchel G, Gordon C, Miller P (1991). The Foucault effect: studies in governmentality.

[74] Haliburton R (2013). Autonomy and the situated self: a challenge to bioethics.

[75] Mackenzie C, Stoljar N (2000). Relational autonomy: feminist perspectives on autonomy, agency, and the social self.

[76] Ives J (2014). A method of reflexive balancing in a pragmatic, interdisciplinary and reflexive bioethics. Bioethics.

