# Supplementary figures and images for: The ECOUTER methodology for stakeholder engagement in translational research
Source: BMC Med Ethics. 2017 Apr 4;18:24. doi: 10.1186/s12910-017-0167-z (PMC5379503; doi:10.1186/s12910-017-0167-z)

## Figure 1: ECOUTER mindmap


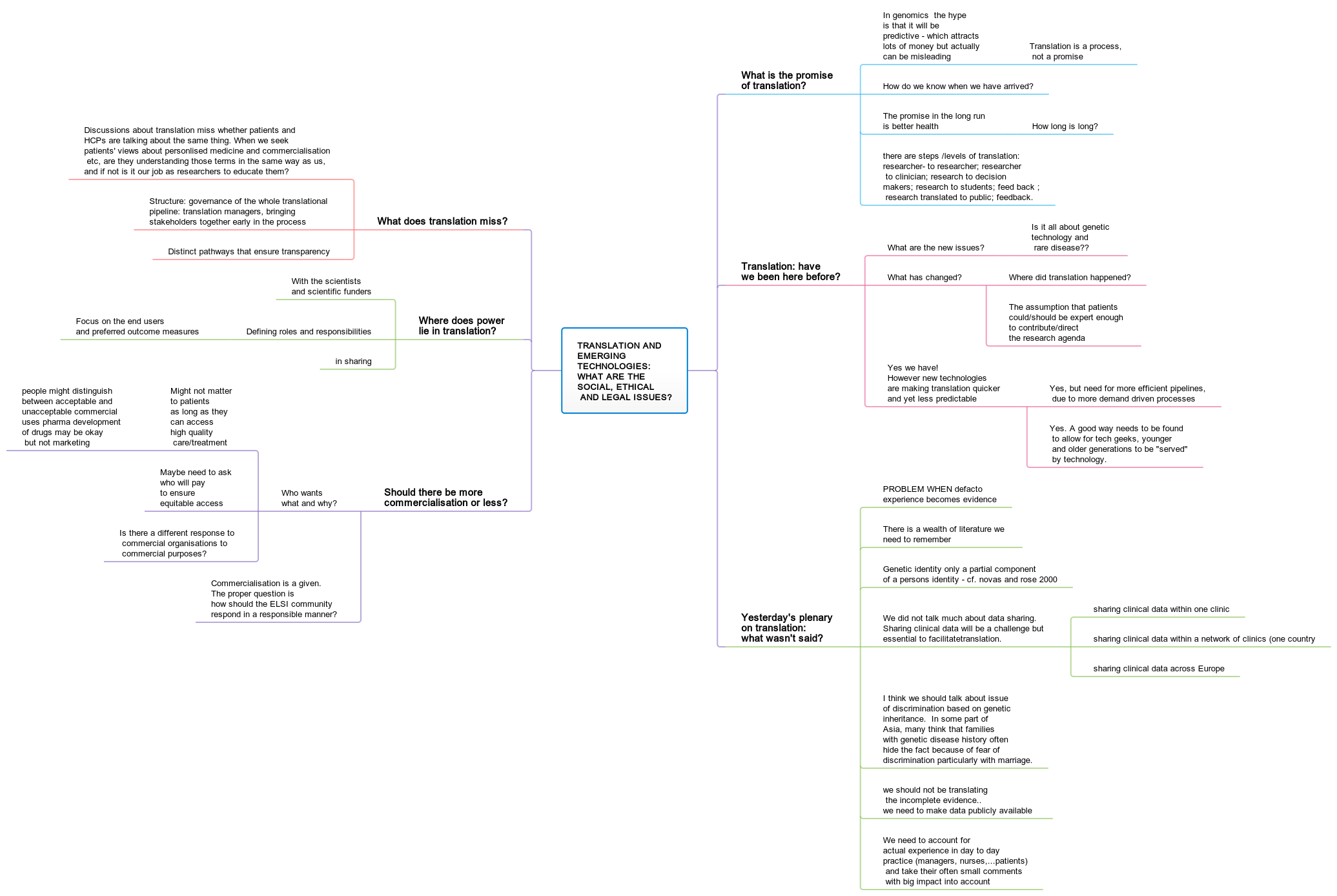

Supplement: Supplementary file 1 — ECOUTER mindmap. Image of final mindmap created during conference ECOUTER event (DOCX 226 kb) [file 12910_2017_167_MOESM1_ESM.docx]
