# Supplementary material for: The ECOUTER methodology for stakeholder engagement in translational research
Source: BMC Med Ethics. 2017 Apr 4;18:24. doi: 10.1186/s12910-017-0167-z (PMC5379503; doi:10.1186/s12910-017-0167-z)
Supplement: Supplementary file 2 — ECOUTER mindmap output. Contributions to ECOUTER mindmap in text form (DOCX 14 kb) [file 12910_2017_167_MOESM2_ESM.docx]

## Appendix 1: ECOUTER mindmap output

- What is the promise of translation?
  - In genomics the hype is that it will be predictive – which attracts lots of money but actually can be misleading
    - Translation is a process not a promise
  - The promise in the long run is better health
    - How long is long?
    - How do we know when we have arrived?
  - There are steps/levels of translation: researcher to researcher; researcher to clinician; research to decision makers; research to students; feedback; research translated to the public; feedback.
- Translation: have we been here before?
  - Yes we have! However new technologies are making translation quicker and yet less predictable
  - Yes, but need for more efficient pipelines, due to more demand driven processes
  - Yes. A good way need to be found to allow for tech geeks, younger and older generations to be “served” by technology
  - What are the new issues?
    - Is it all about genetic technology and rare disease??
  - What has changed?
    - Where did translation happen?
    - The assumption that patients could/should be expert enough to contribute/direct the research agenda
- What does translation miss?
  - Discussions about translation miss whether patients and HCPs are talking about the same thing. When we seek patients’ views about personalised medicine and commercialisation etc., are they understanding those terms in the same way as us, and if not is it our job as researchers to educate them?
  - Structure: governance of the whole translational pipeline: translation managers, bringing stakeholders together early in the process
  - Distinct pathways that ensure transparency
- Where does power lie in translation?
  - With the scientists and the scientific funders
  - Defining roles and responsibilities
    - Focus on the end users and preferred outcome measures
    - In sharing
- Should there be more commercialisation or less?
  - Who wants what and why?
    - Commercialisation is a given. The proper question is how should the ELSI community respond in a responsible manner?
    - Maybe need to ask who will pay to ensure equitable access
    - Is there a different response to commercial organisations? to commercial purposes?
    - Might not matter to patients as long as they can access high quality care/treatment
      - People might distinguish between acceptable and unacceptable commercial uses: pharma development of drugs may be okay but not marketing
- Yesterday’s plenary on translation: what wasn’t said?
  - There is a problem when defacto experience becomes evidence
  - There is a wealth of literature we need to remember
  - Genetic identity only a partial component of a person’s identity – cf Novas and Rose 2000
  - We did not talk much about data sharing. Sharing clinical data will be a challenge but essential to facilitate translation
    - Sharing clinical data within one clinic
    - Sharing clinical data within a network of clinics (one country)
    - Sharing clinical data across Europe
  - I think we should talk about issues of discrimination based on genetic inheritance. In some parts of Asia, many think that families with genetic disease history often hide the fact because of fear of discrimination particularly with marriage
  - We should not be translating the incomplete evidence. We need to make data publicly available.
  - We need to account for the actual experience in day to day practice (managers, nurses, … patients) and take their often small comments with big impact into account.
