# Supplementary material for: The ECOUTER methodology for stakeholder engagement in translational research
Source: BMC Med Ethics. 2017 Apr 4;18:24. doi: 10.1186/s12910-017-0167-z (PMC5379503; doi:10.1186/s12910-017-0167-z)
Supplement: Supplementary file 3 — Analysis of participant contributions in an ECOUTER-first order constructs. Output of first order data analysis (DOCX 17 kb) [file 12910_2017_167_MOESM3_ESM.docx]

## Appendix 2: Analysis of participant contributions in an ECOUTER – first order constructs

*What is the promise of translation?*

In exploring research translation, an obvious first question must be about the definition of the object itself: that is, ‘What is translation?’. Because translation is a self-identified outcome-oriented task, we posed the question on the mindmap, *What is the promise of translation?* In framing the question in this way we deliberately referenced a body of social science literature that offers a critique of both the so-called ‘promise’ of genomics and translation, and of the regimes of hope and promise embedded in contemporary genomics and other ‘translational’ science [74, 75]. In response to this question, participants began to unpick the routine understanding of translation as outcome and to expand the number and type of actors taking part in translation. In their responses participants referenced the framing of translation in terms of ‘promise’, with an early contribution to the mindmap describing the “hype” of genomics and the promise of “predictive” medicine – that the personalised medicine promised by genomics will better ‘predict’ the health outcomes of pharmaceutical therapies for individual patients. This same comment referenced the way the current trend to personalised medicine attracts substantial funding, though the commenter argued that the promise of genomics is “misleading”. To cement this interpretation, another participant made clear this refutation of the ‘promise’ of genomics, stating that ‘translation is a process not a promise’. By highlighting both process and promise, participants were able to disentangle translation as not being merely defined by its outcomes. Though one participant explicitly identified “better health” as the promised outcome of translation, they added the temporal dimension “in the long run”, emphasizing better health as the long term, not necessarily the immediate, outcome or goal of translation. Other participants reinforced this process orientation, deploying spatiotemporal and journey metaphors like “how long is long” and “How do we know when we have arrived?”. This temporal dimension of translation and the theme of process is followed throughout the mindmap. Process was also referenced in the “steps” and “levels” of translation. The seemingly singular definition of translation was made multiple not only in such stages but also in the involvement of numerous actors. Participants posited a range of interpersonal processes of translation: research, publishing and collaboration; communication between researchers, with clinicians, students and public; as part of education; and, through juxtapositioning and interspersing of rounds of ‘feedback’, their positioning in a complex set of relationships.

*“Translation: have we been here before?”*

Our second question deliberately challenged the notion that the ultimate purpose of translation – “better health” – was particularly novel or recent. The advancement of the translational pipeline by the NIH and subsequent explicit emphasis on the outcomes and impact of research in contemporary funding and quality assessment is certainly a recent phenomenon. But is it really the case that medical research prior to the introduction of this concept has been primarily about knowledge for its own sake? Questioning this assumption resonated with participants, and refuting the novelty of translation provided an opportunity to further refine the definition of translation in the contemporary setting. Specifically, participants noted the intensification of the process and the expectations of translation made possible by technology. Here they begin to identify needs and recommendations to address this accelerating process: “more efficient pipelines” (i.e. processes and technologies) accessible to all. Participants raised another important theme for translation: in the call for technology that serves all, non-research and non-medical actors are invoked – e.g. the “younger and older generations” which have been contrasted to the “technology geeks”. Translation was not only about those who are seen to produce health benefit (researchers, clinicians and decision makers), but also explicitly those who may benefit. Responses to the prompt “What has changed?” continued this account by offering an expanded ensemble of actors. One participant explicitly identifies a shift in assumptions about who should direct or shape translation – “... patients could/should be expert enough to contribute/direct the research agenda”. Further resistance to a simplified narrative of translation was also evident: “Is it all about genetic technology and rare disease??”. Together these responses posited translation as a complex process, a practice in which patients and the public may play an active part.

*“What does translation miss?”*

Pushing participants further in their consideration of translation, we asked, *What does translation miss?* The network of relationships and their role in the translational process – the so-called ‘pipeline’ – were explored in response. The description of translation as a complex process necessarily involving a range of actors with a concomitant range of perspectives continued. In this conceptualisation of translation, where patients are one of a number of key actors, questions about the relationships between actors and their understandings of common phenomena were raised. One participant asked whether the actors involved in translation understand technical and other phenomena (e.g. personalised medicine, commercialisation) in the same way: are “patients and HCPs [Health Care Professionals]…talking about the same thing?”. This participant posited education as one remedy. When contrasted against another participant arguing that patients are “expert enough”, this individual appears to imply a deficit model of patient understanding: “is it our job as researchers to educate them?”. Whilst possibly only rhetorical, the question positions patients as lacking knowledge that researchers hold and about which researchers can educate them. Other participants may well have argued against this positioning. Indeed, another individual writing in this node argued for those managing the translation process to bring stakeholders together “early in the process”. ECOUTER participants identified transparency of the processes of translation as crucial and recommended pathways that form the translation process be designed to “ensure transparency”.

*“Where does power lie in translation?”*

Aligned with the quest for transparency above were responses to the trigger question about power, *Where does power lie in translation?* Here, too, there was an expressed desire to include patients as the “end users” of translation. The focus on patients contrasted against those perceived to ‘hold’ power, namely “scientist and scientific funders”. Because we asked the question in terms that assumed power to be hierarchical rather than relational, we unsurprisingly received comments which identified those perceived to be powerful. But this implied definition was resisted in the response “in sharing”, with its inferred collaborative politics.

*“Should there be more commercialisation or less?”*

In the orientation towards inclusivity in the responses above, one type of potential stakeholder is entirely absent: pharmaceutical or other industry. It is hard to know whether this is because translation is perceived as primarily a public enterprise and therefore industry is not considered among the stakeholders of translation, or because we asked about commercialisation, again in a mildly challenging way, in one of the other mindmap nodes. Perhaps, as one participant puts it, “Commercialisation is a given” albeit one that requires responsible and equitable practice and therefore does not merit mention. Talking specifically about commercialisation, participants resisted a simplistic model of commercialisation and attempt to disentangle the question and the concept, distinguishing “commercial organisations” from “commercial purposes” and “acceptable and unacceptable commercial uses”. Orienting their comment to a patient’s possible response, one participant prioritised the outcomes of translation over the means by which it is achieved: commercialisation “might not matter to patients as long as they can access high quality care/treatment”. This use of patient voice undoubtedly has an important rhetorical function. Positioning the statement as a patient response gives it credibility, particularly in the context in which the patient is valorised as the focus of translation and its practice – the leitmotif of this ECOUTER exercise.

*“Yesterday’s plenary on translation: what wasn’t said?”*

Asked to draw out what wasn’t covered in the plenaries on the first day of the conference, participants in the ECOUTER continued along the themes of process, patient and complexity but added other ‘missing’ topics, notably the sharing of data by which translation might be better evidenced: “sharing data within one clinic”, “a network of clinics (one country)” and “across Europe”. This call to share data and make “data publicly available” was then reinforced by another participant who noted the need to translate all the available evidence, not “incomplete evidence”, and referenced a positive science in which such complete evidence, and its corollary complete translation, is a possibility. This reference to the science of translation is a minor theme in comparison to a predominant focus on patients and other stakeholders, and is interesting in and of itself for its contrast with most discussions of translation in the literature where the opposite is true. Indeed, we were asked by one participant not to forget the wealth of other literature, which they described in a micro-discussion as the social science literature on genomics and translation and later reference on the mindmap through a paper by Novas and Rose [76] to evidence their point about identity and genomics.

Other comments about what was missed in the plenaries followed the theme of disentangling the subjects of translation and highlighting the complexity of issues of identity that are raised: a plea not to conflate identity and genomics, discrimination based on genetic identity, and the context of culture in the meaning of identity and genomics. Two comments about experience appeared to contradict one another but as so often with negative cases reinforced the argument presented in the ECOUTER as a whole. One comment argued the need to take account of the lived experience of patients, nurses, managers, etc., giving as an example the often small changes noticed only through direct experience in healthcare practice that can have a “a big impact” on patients’ outcomes. For instance, the greatly increased quality of life offered by largely inexpensive tools for communication (e.g. accessibility apps for those with conditions affecting motor coordination) were compared with expensive drugs that provide only marginal improvement in dexterity. This participant argued that learning from these seemingly small changes and the lived-experience of healthcare practitioners and patients should not be obscured by the lure of expensive translational research. Another individual, apparently in contrast, identified “a problem when defacto experience becomes evidence”. They argued separately in a micro-discussion with one of the D2K facilitators that the particular experiences of some patients (e.g. those with rare diseases referenced in one plenary) should not be taken to represent the experience of all patients. Rather than a refutation of the importance of patient experience, they argued for an evidenced (qualitative) understanding of experience, one which assumed variation rather than singularity of experience and therefore of perspectives. Though at face value contradicting one another, these two comments reinforced not only the importance of considering the complexity of healthcare systems and the translation of research into practice, but equally the importance of taking seriously the voice of the range of stakeholders, especially patients and those at the coalface of healthcare practice.

**References**

74. Brown N, Michael A. A sociology of expectations: retrospecting prospects and prospecting retrospects. Technol Anal Strateg. 2003;15:3–18.

75. Martin P, Brown N, Turner A. Capitalizing hope: the commercial development of umbilical cord blood stem cell banking. New Genet Soc. 2008;27:127–43.

76. Novas C, Rose N. Genetic risk and the birth of the somatic individual. Econ Soc. 2000;29:485–513.
