# Supplementary material for: The ECOUTER methodology for stakeholder engagement in translational research
Source: BMC Med Ethics. 2017 Apr 4;18:24. doi: 10.1186/s12910-017-0167-z (PMC5379503; doi:10.1186/s12910-017-0167-z)
Supplement: Supplementary file 4 — ECOUTER conceptual schema-second order constructs. Description of data: Output of second order data analysis (DOCX 13 kb) [file 12910_2017_167_MOESM4_ESM.docx]

## Appendix 3: ECOUTER conceptual schema – second order constructs

***‘Perspectives’*** revealed the ways in which delegates sought to expand consideration of translation and open it up to critique. Contributions to the ECOUTER revealed widespread efforts by delegates to reflect critically on translation and open concepts discussed by speakers up to further examination. The engagement process afforded delegates a forum in which to challenge ideas and highlight values they saw as integral to understanding the field. Central to this process was an effort to widen our understanding of translation by moving beyond the confines of academia, integrating more fully a wider variety of worldviews and real life situations (including how research is conducted). Delegates were interested in posing questions intended to push boundaries and move beyond current scholarly frameworks. Underscoring many of the contributions was a sense of resistance, of gently pushing back at the dominant rhetoric of translation. Delegates contested the ways translation is being and has been understood and approached; such contributions were seen to explore the limitations of translational efforts and the values that appear to drive current thinking. In this way, delegates were in effect asking dominant stakeholder communities (namely scholars and researchers) to reflect more comprehensively and critically on what questions can and should be asked of translation in order to challenge prevailing discourses.

***‘Process’*** spoke to those values delegates appreciated as essential to how translation was made to happen, including a need to open up translational research and technology to greater scrutiny. If delegates were interested in challenging current perspectives, they were equally intrigued by how translation actually happens. This was well illustrated by delegates who expressed a need to open up the “black box of research” (e.g. in the context of senior researchers and funders who drive the politicised process of critical discussion in which junior researchers typically have little or no voice). Using the ECOUTER process delegates were able to voice the perspective that no aspect of translation should remain routinely overlooked or under-examined. Transparency and openness were seen as critical not only for technology and infrastructure, but for relationships between individuals. Personal connections were essential to the ‘humanness’ that drives the promises and expectations of the translation process. The process of exchange (e.g. in ECOUTER and other engagement) was identified as holding elemental value in opening up the translation process, with delegates emphasising the degree to which exchange allowed translation to take place. Indeed, the social value of exchange ultimately made possible the aim and expectation of the translation process.

***‘People’*** emphasised that translation was first and foremost about relationships and understanding such connections between individuals across the translation spectrum from development of knowledge and its translation into practice was foundational. Delegates were able to highlight using the ECOUTER the degree to which translation was ultimately a socially enacted phenomenon. At its core, translation was seen to be grounded in people, their actions (individual and collective) and the relationships they form. And, if at its core translation in healthcare is a ‘peopled’ process, it must be understood ethnographically. Delegates posited multiple voices – or peoples – in translation: many of whom remain unheard.
